# Without a body of evidence and peer review, taxonomic changes in Liolaemidae and Tropiduridae (Squamata) must be rejected

**DOI:** 10.3897/zookeys.813.29164

**Published:** 2019-01-07

**Authors:** Jaime Troncoso-Palacios, Margarita Ruiz De Gamboa, Roberto Langstroth, Juan Carlos rtiz, Antonieta Labra

**Affiliations:** 1 Programa de Fisiología y Biofísica, Facultad de Medicina, Universidad de Chile, Independencia 1027, Casilla 70005, Santiago, Chile Universidad de Chile Santiago Chile; 2 Centro de Investigación en Medio Ambiente (CENIMA), Universidad Arturo Prat, Casilla 121, Iquique, Chile Universidad Arturo Prat Iquique Chile; 3 Colección Boliviana de Fauna, Calle 27 Cota Cota, La Paz, Bolivia Colección Boliviana de Fauna La Paz Bolivia; 4 Departamento de Zoología, Facultad de Ciencias naturales y Oceanográficas, Universidad de Concepción, Casilla 160-C, Concepción, Chile Universidad de Concepción Concepción Chile; 5 Centre for Ecological and Evolutionary Synthesis (CEES), Department of Biosciences, University of Oslo, P.O. Box 1066 Blindern, Norway University of Oslo Oslo Norway

**Keywords:** Taxonomy, best practices, International Code of Zoological Nomenclature, synonymy, *
Liolaemus
*, *
Microlophus
*, *
Phymaturus
*

## Abstract

In his recent self-published book "Reptiles en Chile", Diego Demangel Miranda presented 13 taxonomic changes for liolaemid and tropidurid lizards. While these could be considered validly published according to the International Code of Zoological Nomenclature, we show that these taxonomic propositions lack the necessary scientific rigor in terms of replicability, specimen work, lack of peer review and that they do not follow best practices accepted by the herpetological community. Therefore, we hereby invalidate all 13 taxonomic changes proposed in this book, leaving the taxonomy unaffected. Finally, we call attention to the potentially negative consequences of using these taxonomic changes in conservation and environmental impact studies as incorrect decisions might be taken in relation to the species involved.

## Introduction

Field guides are a common source of information for the general public interested in the identification of plants and animals in a region. However, popular guidebooks are not intended to be vehicles for taxonomic decisions and should follow the taxonomy supported by the most recent peer-reviewed scientific studies. A reliable taxonomy, the science of describing, naming, and/or synonymizing taxa (Enghoff and Seberg 2006), requires multiple lines of evidence (e.g., de Queiroz 1998) supported by the appropriate comparisons of voucher specimens (Ceríaco et al. 2016). Furthermore, any such study needs to be submitted to the opinions and comments of other experts; thus, taxonomic studies should be published only after peer review (Kaiser et al. 2013; Schutze et al. 2017). Whereas the International Code of Zoological Nomenclature (ICZN 1999; hereafter the Code) regulates the nomenclatural acts, it does not have the aim of regulating the methods by which taxonomic decisions should be generated and how any resulting change should become part of the scientific record (Kaiser 2013). This situation allows publication of taxonomic decisions in a non-scientific manner, with insufficient or unsuitable methodology, with lack of evidence, or via self-publication without peer review. In many cases, these unscientific changes are numerous and have affected several taxonomic groups, as has been carefully critiqued (Jäch 2007a, b; Wallach et al. 2009; Kaiser 2013, 2014a; Reynolds et al. 2014; Rhodin et al. 2015). The most controversial cases in herpetology are the names created by Raymond Hoser, who self-publishes the Australasian Journal of Herpetology (see Kaiser et al. 2013). Hoser’s publications (e.g., Hoser 2012a, b) may include the basic requirements for valid nomenclature, such as availability of the publication, names for proposed taxa, holotype for species descriptions and diagnoses for proposed taxa (Wüster et al. 2001). However, these are provided in unacceptable ways, without proper evidence or peer review and sometimes with substantial plagiarism and ethical breaches (Kaiser et al. 2013; Denzer et al. 2015; Rhodin et al. 2015). While one case regarding Hoser’s self-published names is pending a ruling by the International Commission on Zoological Nomenclature (e.g., Kaiser 2014b; Rhodin et al. 2015), nine herpetologists, supported by 100 other herpetologists as well as several large herpetological societies and journals, have determined that unscientific taxonomic decisions in herpetology without a body of evidence and without peer review should be unacceptable for the purposes of herpetological taxonomy (Kaiser et al. 2013).

Recently, Diego Demangel Miranda published the book "Reptiles en Chile" (Demangel Miranda 2016a), which is an exceptional source of high-quality photographs of living Chilean non-avian reptiles, especially relevant for species without previously published photographs. However, the book includes several taxonomic changes in the Liolaemidae and Tropiduridae along with several changes in the geographic distribution for various species. Here, we examine whether these taxonomic changes fulfill the currently recognized best practices in herpetology (Kaiser et al. 2013).

## Features of "Reptiles en Chile" by Demangel Miranda (2016a)

The 619-page book starts with 20 pages of general overview (cover page, acknowledgments, prologue, preface, and presentation). Thereafter, it provides generalities about reptiles, as well as some information of their characteristics, evolution, and species concepts (pp. 21–39). It then provides a list of Chilean reptile species accepted by the author (pp. 40–44), and guidelines to facilitate the identification of the Chilean reptile groups in the field (pp. 45–50). Thereafter (pp. 51–59), the book gives data on biogeography and species conservation, followed by a glossary (pp. 60–63), and an explanation (pp. 64–65) of how to use the book according to the information provided for each species.

**Figure 1.**
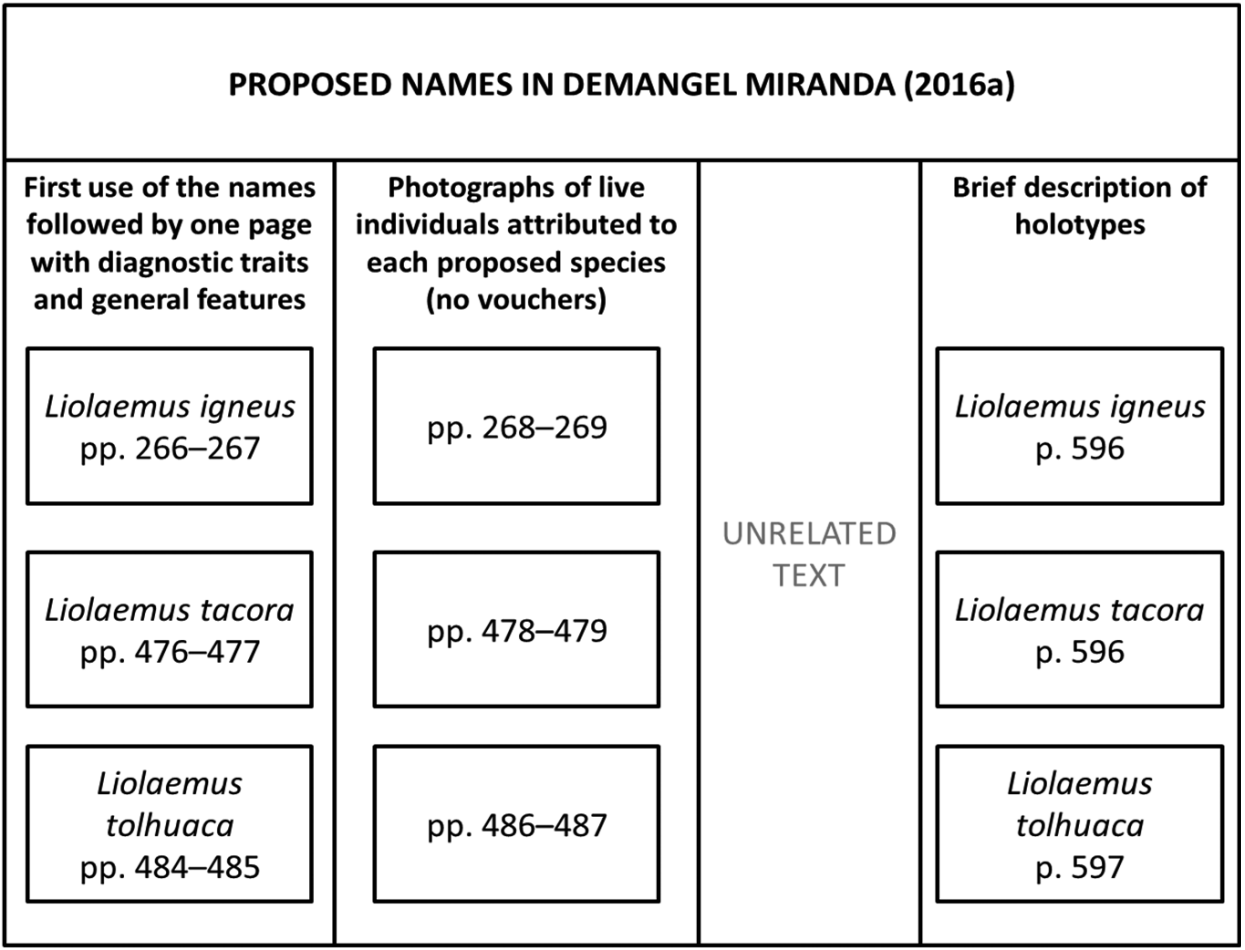
Outline of the non-standard presentation of the three species proposed by Demangel Miranda (2016a).

The bulk of the book (pp. 66–583) provides accounts of each species, including (for almost all species) three pages of photos of live individuals attributed to the species (each indicating the locality). Each species account begins with a caption indicating the species name, authority, and, in some cases, a list of “formal synonyms or other scientific names used in the last 30 years” (our translation, p. 64). However, the synonymies do not indicate the authority or year. Each species account has one page with text, including the etymology of the name, type locality, diagnosis, morphological features, distribution, natural history notes, some specific references, as well as a distribution map and usually three small photos. In total, this section has 143 pages of text (i.e., the text occupies a column, with maps and species photos occupying another column) and 375 pages of photos. Remarkably, the species accounts include three species names proposed by the author but following an unusual presentation for taxonomic descriptions. While standard taxonomic publication introduces the first use of the name and then presents the diagnostic traits and description of the holotype and other types in consecutive pages (e.g., Quinteros et al. 2014), Demangel Miranda (2016a) provided the first use of the name and the diagnostic features on consecutive pages of the book, but placed the brief holotype descriptions at the end of the book in a section entitled “Taxonomic notes” (pp. 584–597, Fig. 1).

The taxonomic notes section includes the changes proposed in the book: one for Tropiduridae (one synonymy) and 12 for Liolaemidae (nine synonymies and three proposed species). On pages 596–597, the author lists the type specimens for the three proposed species and provides a brief description of the holotypes. These two pages of descriptions also include three small photographs displaying the three holotypes in life as well as a photo of two live individuals (placed on the same rock), stating that these are individuals of two of the described species. Finally, the literature cited in Demangel Miranda’s book is presented on pp. 598–612, followed by the name index (pp. 614–618).

Although Demangel Miranda (2016a) included several taxonomic decisions, there is no section describing the methods he used to arrive at these decisions. Such a section is fundamental in any scientific study, including, for example, other recent descriptions of Chilean *Liolaemus* (e.g., Núñez et al. 2000; Valladares 2004; Esquerré et al. 2013; Quinteros et al. 2014). While there are some statements in Demangel Miranda (2016a) which indicate that the author performed “scale counts,” he did not describe how these were done. Beyond the five specimens used for the descriptions of his three proposed species, Demangel Miranda (2016a) stated that he examined the holotype of *L.lopezi* Ibarra-Vidal, 2005 (p. 586), one specimen of *L.molinai* Valladares et al., 2002 (p. 590), two specimens of *L.frassinettii* Núñez, 2007 (p. 590), one specimen of *L.carlosgarini* Esquerré et al., 2013 (p. 592), and two syntypes of *L.melanopleurus* (Philippi, 1960) (p. 592). These 12 specimens are not listed in an appendix of material examined, contrary to standard practice in taxonomy (e.g., Lobo and Espinoza 2004; Abdala and Quinteros 2008; Breitman et al. 2011b; Avila et al. 2012). Furthermore, Demangel Miranda (2016a)also stated that he examined the type series of *L.velosoi* Ortiz, 1987, and *Microlophustarapacensis* (Donoso-Barros, 1966) at the MZUC (Museo de Zoología de la Universidad de Concepción), but he did not provide catalog numbers. He also indicated that he reviewed MZUC specimens labeled as *L.brattstroemi* Donoso-Barros, 1961, indicating that these were not the types, and that he instead assigned these to *L.cyanogaster* (Duméril & Bibron, 1837), but he, again, did this without providing catalog numbers or data to support this claim. This lack of examined material is a serious problem of the proposed taxonomic changes included in this book.

Copies of Demangel Miranda’s book were first available on 30 June 2016 (2000 copies). We remark that some copies were sold before the launching (pers. obs.). The book was published by Fauna Nativa Ediciones and, to our knowledge, this is the first and sole book published by this company. Moreover, Fauna Nativa Ediciones is the publishing arm of Fauna Nativa Consultores SPA (http://www.faunanativa.cl), which is a company owned by Diego Demangel Miranda dedicated to wildlife evaluation services in regard to environmental impact studies. No editor is mentioned by Demangel Miranda (2016a), but the verso of the title page indicates “Texts”, “Photographs” and “Photographic edition” by Diego Demangel and “Style revision”, “General design”, “Graphical design”, and “Layout” are credited to other people. Thus, it can be concluded that Diego Demangel Miranda was solely responsible of the final review of the text (i.e., acted as editor) and that he took all decisions in relation to the taxonomic changes made.

## Taxonomic changes proposed in Demangel Miranda (2016a)

Demangel Miranda (2016a) started the book indicating that “… [this] is not a conventional scientific work” (p. 17), which may cause some uncertainties for the reader. Then, in the first paragraph of the section where the taxonomic changes are presented (pp. 584–597), Demangel Miranda stated that “…it was not possible to review all the literature regarding the different species and therefore many readers may be disappointed with the text in their hands…” (our translations, p. 584) which also may cause some uncertainties. The 13 taxonomic changes made by Demangel Miranda (2016a) in Liolaemidae and Tropiduridae are summarized in Table 1.

**Table 1.** Summary of the taxonomic changes proposed by Demangel Miranda (2016a). Details of recommendation are explained in the text. *Liolaemus* subgenera are according to Abdala and Quinteros (2014).

Family	Genus / Subgenus (if correspond)	Taxon	Demangel Miranda (2016a) proposed	Recommendation
Liolaemidae	*Liolaemus* / *Liolaemus* (sensu stricto)	*Liolaemusbrattstroemi* Donoso-Barros, 1961	Synonym of *Liolaemuspictus* (Duméril & Bibron, 1837)	Not acceptable
		*Liolaemuschungara* Quinteros, Valladares, Semham, Acosta, Barrionuevo & Abdala, 2014	Synonym of *Liolaemusalticolor* Barbour, 1909	
		*Liolaemuskuhlmanni* Müller & Hellmich, 1933	Synonym of *Liolaemuszapallarensis* Müller & Hellmich, 1933	
		*Liolaemusvelosoi* Ortiz, 1987	Synonym of *Liolaemusplatei* Werner, 1898	
	*Liolaemus* / *Eulaemus*	*Liolaemuslopezi* Ibarra-Vidal, 2005	Synonym of *Liolaemusornatus* Koslowsky, 1898	
		*Liolaemusmorandae* Breitman, Parra, Pérez & Sites, 2011	Synonym of *Liolaemuslineomaculatus* Boulenger, 1885	
		*Liolaemusscolaroi* Pincheira-Donoso & Núñez, 2005	Synonym of *Liolaemuszullyae* Cei & Scolaro, 1996	
	* Liolaemus *	* Liolaemus igneus *	Proposed species	
		* Liolaemus tacora *	Proposed species	
		* Liolaemus tolhuaca *	Proposed species	
	* Phymaturus *	*Phymaturusaguedae* Troncoso-Palacios & Esquerré, 2014	Synonym of *Phymaturusdarwini* Núñez, Veloso, Espejo, Veloso, Cortés & Araya, 2010	
		*Phymaturusdamasense* Troncoso-Palacios & Lobo, 2012	Synonym of *Phymaturusmaulense*, Núñez, Veloso, Espejo, Veloso, Cortés & Araya, 2010	
Tropiduridae	* Microlophus *	*Microlophusyanezi* (Ortiz, 1980)	Synonym of *Microlophustheresioides* (Donoso-Barros, 1966)	

The main failure of the taxonomic changes proposed by Demangel Miranda (2016a) is that these were not published via peer review. As demonstrated above, he was both his own editor and the owner of the publisher which produced the book. Although Demangel Miranda (2016a, p. 6) stated that Juan Carlos Torres-Mura, a Chilean zoologist, reviewed “some sections of the book”, this cannot be considered a peer review because Torres-Mura reviewed only “part of the text” and it is unclear which sections. Moreover, in a scientific publication the author does not choose the reviewers (while some journals allow suggesting reviewers, which is different than choosing) and because Demangel Miranda himself is credited as responsible for all texts (editor), this procedure cannot be considered to fulfill the objectives of an appropriate peer review (see Voight and Hoogenboom 2012). As recommended by Kaiser et al. (2013), peer review should involve at least two independent reviewers and an editor who can objectively be considered experts in the field of the manuscript under review. This lack of peer review is a strong argument to indicate that the taxonomic acts in Demangel Miranda (2016a) should not be accepted by the herpetological community.

In the following paragraphs, we provide additional information to support our conclusions that Demangel Miranda (2016a)also failed to meet the accepted best practice in herpetological taxonomy. There is no “body of evidence” to support his synonymies and proposed species, which could have been done by using the available literature and by appropriate data analysis, as is required by best practices (Kaiser et al. 2013).

### Synonymies

One general problem is that Demangel Miranda (2016a) did not refer to material examined for the junior or senior synonyms proposed, apart from the holotype of *L.lopezi* (all other specimens listed are not involved in the taxonomic changes). This omission is problematic as the examination and listing of specimens are key aspects of correct taxonomic practice (see Dubois 2017a), which allows others to build knowledge based on the new data. Moreover, the lack of a section describing how the synonymies were developed or the proposed species were described (i.e., lack of materials and methods) makes the conclusions reached by Demangel Miranda (2016a)a non-replicable result.

### *Liolaemus brattstroemi*

Demangel Miranda (2016a, pp. 394, 590–591) declared *Liolaemusbrattstroemi* Donoso-Barros, 1961 to be a junior synonym of *L.pictus* (Duméril & Bibron, 1837). However, he provided no comparative data for *L.pictus* (neither from reviewed vouchers nor references) to support this claim. The author only supported this proposed synonymy by a visit to the type locality of *L.brattstroemi*, where he found lizards that he considered assignable only to *L.pictus*, without indication of how many individuals were analyzed to reach this conclusion or provide a reliable data analysis.

### *Liolaemus chungara*

Demangel Miranda (2016a, pp. 142, 591) included *Liolaemuschungara* Quinteros et al., 2014, as a junior synonym of *L.alticolor* Barbour, 1909. Demangel Miranda (2016a, p. 591) proposed this synonymy based on the presence and absence of precloacal pores in the males he found during a field trip to the type locality of *L.chungara*. However, there is no indication of how many males were sampled and no information on the examined voucher specimens (neither of *L.chungara* nor of *L.alticolor*) was provided. In addition, the accuracy of the determination of the precloacal pores in the field remains unclear, considering that an appropriate observation of these types of pores requires the use of magnifying lenses, whose use was not indicated by Demangel Miranda (2016a). Moreover, it was not indicated how he concluded that all the observed males were of the same species or how he concluded that this supposed variation is “a relatively common feature” in *L.alticolor*.

### *Liolaemus lopezi*

Demangel Miranda (2016a, pp. 374, 585–586) included *Liolaemuslopezi* Ibarra-Vidal, 2005, as a junior synonym of *L.ornatus* Koslowsky, 1898. In contrast to the procedure used to propose the other synonymies in his book, in the case of *L.lopezi* Demangel Miranda examined the holotype of this species (Museo Regional de Concepción, CHMHNC 1099); however, he did not review vouchers of *L.ornatus*.

He cited Pincheira-Donoso and Núñez (2005) as his only source of data for *L.ornatus* morphological variation (p. 585) and despite being unsure if these data really belong to *L.ornatus* (p. 586), he still proposed the synonymy.

### *Liolaemus kuhlmanni*

Demangel Miranda (2016a, pp. 512, 592) included *Liolaemuskuhlmanni* Müller & Hellmich, 1933, as a junior synonym of *L.zapallarensis* Müller & Hellmich, 1933, as previously proposed by Pincheira-Donoso and Núñez (2005) but rejected by Lobo et al. (2010a). Demangel Miranda (2016a) proposed this synonymy without providing information on the specimens analyzed (i.e., vouchers reviewed) or any other supporting evidence (e.g., data of compared scale count ranges or morphological measures, statistical analysis, molecular data). While referring to Lobo et al. (2010a) in an unrelated paragraph (e.g., p. 590), Demangel Miranda (2016a) did not mention that the same study had rejected the prior synonymy of *L.kuhlmanni* under *L.zapallarensis*. This procedure clearly did not fulfill “the third line of evidence” that a reliable taxonomic study needs to follow (Kaiser et al. 2013, p. 18), because there is an important omission of a key published scientific study that must have been included in the “body of knowledge” on *L.kuhlmanni*.

### *Liolaemus morandae*

Demangel Miranda (2016a, pp. 306, 586) included *Liolaemusmorandae* Breitman et al., 2011 as a junior synonym of *L.lineomaculatus* Boulenger, 1885. Breitman et al. (2011b) split *L.lineomaculatus* into three species: *L.morandae*, *L.avilae*, and *L.lineomaculatus* (with allopatric distributions from north to south, respectively), based on a principal component analysis (PCA), a multivariate analyses (MPMANOVA), and a multilocus phylogeny with examination of 36 specimens of these species. Demangel Miranda (2016a, p. 586) proposed the synonymy based on comparisons of live individuals that he found during field trips to Aysén and Magallanes Regions, Chile, but without inclusion of animals from the type locality of *L.morandae*. He claimed that “the diagnosis provided by Breitman et al. (2011b) does not allow a proper separation between *L.morandae* and *L.lineomaculatus*” (Demangel Miranda 2016a, p. 586), without mention *L.avilae*. Moreover, he pointed out that an integrative taxonomic study should be performed to evaluate if it is appropriate to split *L.lineomaculatus* into species or subspecies, without acknowledging the study already published by Breitman et al. (2011b). While best practices indicate the need for “rigorous” taxonomic analyses (Kaiser et al. 2013, p. 8), Demangel Miranda (2016a) failed in regard to this synonymy due to his total lack of evidence to refute the results of Breitman et al. (2011b), which is indeed an integrative taxonomy study in *Liolaemus*.

### *Liolaemus scolaroi*

Demangel Miranda (2016a, pp. 516, 585) included *Liolaemusscolaroi* Pincheira-Donoso & Núñez, 2005 as a junior synonym of *L.zullyae* Cei & Scolaro, 1996. Demangel Miranda (2016a) based his synonymy on a field trip to the type locality of *L.scolaroi*, during which he claims to have examined live individuals, but without providing supporting data, analysis, and results. Moreover, Demangel Miranda (2016a) did not refer to the previous publications (Breitman et al. 2011a, 2014) that had already suggested this possible synonymy.

### *Liolaemus velosoi*

Demangel Miranda (2016a, pp. 398, 593) included *Liolaemusvelosoi* Ortiz, 1987, as a junior synonym of *L.platei* Werner, 1898. Although Demangel Miranda (2016a) stated that he reviewed the *L.velosoi* type series, he did not provide catalog numbers and did not mention examining any voucher specimens of *L.platei*. Demangel Miranda (2016a) did not provide any data for the scale counts considered diagnostic for these species (Ortiz 1987; Pincheira-Donoso and Núñez 2005) or other type of evidence apart from the photos in which he stated that the color variation overlaps between species. The mtDNA phylogeny of Troncoso-Palacios et al. (2015) showed a deep genetic divergence between these species, but this was not taken in account by Demangel Miranda (2016a)as part of the body of knowledge for this species.

### *Phymaturusaguedae* and *P.damasense*

Demangel Miranda (2016a, pp. 530, 534, 593) included *Phymaturusaguedae* Troncoso-Palacios & Esquerré, 2014, as a junior synonym of *P.darwini* Núñez et al., 2010, and included *P.damasense* Troncoso-Palacios & Lobo, 2012 as a junior synonym of *P.maulense* Núñez et al., 2010. Demangel Miranda (2016a) based both synonymies on live individuals (no vouchers were listed) that he observed in field trips, but in both cases he did not list all the visited localities and he only provided the same ambiguous sentence for both synonymies: “[I] have carried out multiple field trips to the localities where [these *Phymaturus*] are known”. He also indicated that “an inter-population analysis was performed” without indicating the methodology, data or results of this analysis. Additionally, he stated that he compared scale counts and scale sizes (i.e., preocular and canthal) on live individuals, without indication of how many individuals were examined. It should be indicated that scales in *Phymaturus* are very small and taxonomic studies performed in this genus have declared the use of magnification lenses for proper observation (e.g., Lobo et al. 2010b), which suggest that field observations of scales made by Demangel Miranda are neither rigorous, appropriate, nor reliable.

### *Microlophus yanezi*

Demangel Miranda (2016a, pp. 560, 595) included *Microlophusyanezi* (Ortiz, 1980) as a junior synonym of *M.theresioides* (Donoso-Barros, 1966). Ortiz (1980) described *M.yanezi* and distinguished it from *M.theresioides* based on the average counts of midbody scales and average counts of scales in the fourth toe lamellae. However, Demangel Miranda (2016a) only compared the ranges of these scale counts based on Ortiz (1980), without vouchers or another data source, and without mention of the average differences, or additional evidence to refute Ortiz’s (1980) conclusions. Moreover, without evidence as in the case of *L.morandae*, Demangel Miranda (2016a)attempted to undermine a previous published scientific study.

### Species names proposed by Demangel Miranda (2016a)

Demangel Miranda (2016a) proposed three names: *Liolaemusigneus* (p. 266), *L.tacora* (p. 478) and *L.tolhuaca* (p. 486), with small sample sizes (*n* = 3, *n* = 1 and *n* = 1, respectively) and through an odd presentation that does not follow the standard taxonomic descriptions (see Fig. 1). The holotypes were placed in the Museo Nacional de Historia Natural de Chile. All the proposed names lack a hypothesis of group membership apart of from being assigned to the genus *Liolaemus*, a very diverse genus (257 species; Abdala and Quinteros 2014) which includes two well-supported subgenera, each composed of several groups (see Lobo et al. 2010a; Abdala and Quinteros 2014). However, none of the species proposed by Demangel Miranda (2016a) were assigned to either of these subgenera. This omission is a failure to fulfill the first step of the best practices in herpetology when species are described (Kaiser et al. 2013, p. 8).

Moreover, Demangel Miranda (2016a) did not provide analyses and appropriate methodology to support his species hypotheses. For example, the “diagnostic features” were based solely on some color patterns, and shape and size of some scales. When the author attempts to utilize the size of the specimens as diagnostic feature, he only used ambiguous comparative expressions such as “bigger than” or “smaller than”. Although *Liolaemus* comprises some 257 species, Demangel Miranda (2016a) precariously compared his three proposed species with only five *Liolaemus* species (without indication of reviewed vouchers): he only compared *L.igneus* and *L.tacora* against *L.jamesi* and with each other; he compared *L.tolhuaca* against only four other *Liolaemus* (*L.buergeri*, *L.lonquimayensis*, *L.scorialis* and *L.zabalai*). This is worsened by the fact that Demangel Miranda (2016a) failed to provide evidence of the supposed species as cohesive populations, which is necessary according to the best practices (Kaiser et al. 2013, p. 18). For example, *L.igneus* is based on three specimens collected at three different localities (p. 596), without evidence to support that these three specimens conformed a recognizable cohesive population assignable to a species. Moreover, *L.tacora* and *L.tolhuaca* are based only on one specimen each (pp. 597–98), but despite this, he indicated variation for some features (e.g., scale count ranges, differences between males and females) without reference to any paratypes or other material examined or reference (i.e., the source of the variability of his data is unknown). Remarkably, for *L.igneus* and *L.tacora*, Demangel Miranda (2016a, p. 267 and p. 477, respectively) included a “species bibliography” listing “Abdala et al. (2008)”, “Quinteros et al. (2008)”, and “Quinteros and Abdala (2011)”, but there are neither indications of the aim of these references in the context of the characterizations nor their inclusion in the book bibliography. We can only speculate that these may refer to studies describing Argentine *Liolaemus* species. These facts allow us to conclude that Demangel Miranda (2016a) did not fulfill the second step of the best practices, to test the taxonomic hypothesis through a “rigorous, honest, and appropriate methodology” (Kaiser et al. 2013, p. 8).

As previously pointed out, the major problem with the three species names proposed by Demangel Miranda (2016a)and all his other taxonomic decisions is that they were not published via peer review, which is in opposition to the third step of the best practices proposed by Kaiser et al. (2013, p. 8).

Finally, Demangel Miranda (2016a) does not denote an effort to maintain taxonomic stability in *Liolaemus*, *Phymaturus* and *Microlophus*, as he proposed 13 taxonomic decisions for two squamate families, with very few reviewed vouchers and without reliable analyses, and rather represented his own vision in an authoritarian way, which is not in agreement with the appropriate practices in taxonomy (Kaiser et al. 2013, Schutze et al. 2017).

## Final remarks

In addition to the taxonomic instability produced by the propositions already discussed, the taxonomic and distributional changes performed in the book may potentially have major negative consequences affecting society at large. The use of this type of unreliable taxonomy by agencies or institutions dealing with biodiversity and conservation problems can lead to incorrect decisions with potential negative consequences such as the loss of biological resources (Wilson 1985; Pillon and Chase 2007; Georges et al. 2011; Kaiser et al. 2013; Thomson et al. 2018). Although the last updated reptile list for Chile (Ruiz de Gamboa 2016) did not consider Demangel Miranda’s (2016a) taxonomic changes because of the “lack of scientific rigor” in it, Demangel Miranda (2016a) has already begun to be used by the Chilean government agencies that deal with conservation and biodiversity. For example, "Vertebrados en Peligro de la Región Metropolitana de Santiago, Chile" [Endangered Vertebrates from the Metropolitan Region of Santiago, Chile] by Carrasco-Lagos et al. (2016) and the background record for conservation purposes of *Hydrophisplaturus* (Tala, 2016), both published by the Chilean Ministry of the Environment, cite Demangel Miranda (2016a). Demangel Miranda (2016a)also is cited in environmental documents involved in the approval of the proposal of a power generation plant submitted to the Chilean Environmental Impact Assessment System (file N° 52, http://seia.sea.gob.cl/expediente/expedientesEvaluacion.php?modo=ficha&id_expediente=2131347751). The problem is that while Demangel Miranda (2016a) compiled useful information, at the same time the publication changes several important aspects for numerous Chilean lizard species without scientific support (e.g., distributional ranges and taxonomy). Remarkably, the three names proposed and two of the synonymies (*L.kuhlmanni* and *P.damasense*) by Demangel Miranda (2016a) have been also followed by the widely used “Reptile Database” (Uetz et al. 2018, accessed in December 3, 2018), without indication of why these two synonymies are followed whereas the other are not.

The book also generates confusion about what is a taxonomic synonym. The listing of names as synonyms by Demangel Miranda (2016a) does not follow the accepted practice (e.g., names do not include any author citations or year), which could lead to incorrect interpretations. For example, he recognizes *L.islugensis* and L.cf.pantherinus as full species, but at the same time, includes each in the synonymy of the other (pp. 274, 378).

Later in 2016, Demangel Miranda launched a second field guide entitled "Reptiles del Centro Sur de Chile" (Demangel Miranda 2016b). This new book is a simplified version of Demangel Miranda (2016a) that covers only the species of central and southern Chile and does not include species descriptions or new synonymies. The guide reflects the taxonomic and distributional changes of Demangel Miranda (2016a), hence, we recommend to not follow it as taxonomically reliable source.

Finally, concerning the Code, Article 8.1 requires that for a study to be considered as published for the purposes of zoological nomenclature “it must be issued for the purpose of providing a public and permanent scientific record”. The problem here is that the Code does not define “scientific record”. Demangel Miranda (2016a) himself declares that “the book is not a conventional scientific publication” (p. 17), as is evidenced by the lack of several sections and procedures typically used in scientific publications such materials and methods, analyses of the characters and publication via peer review. For us, it is clear that he did not intend the book to be a scientific review of Liolaemidae and *Microlophus*, but rather the purpose was the diffusion of knowledge of the Chilean reptile for a wide range of Chilean readers. Some authors have stated that the names proposed in publications that are not intended to be “scientific record” should be considered as not valid nomenclatural act (e.g., Busack et al. 2016), a detailed clarification of this issue is provided by Schleip (2014); if this strict interpretation of the Art. 8.1 is applied to Demangel Miranda (2016a), the three names proposed could be considered as not valid, but we think that the sole publication of these names without fulfilling the best practices (Kaiser et al. 2013) should be enough to avoid their use. Additionally, we suggest that Art. 13.1 of the Code, needs to be improved because this requires that the description must include the characters “purported to differentiate the taxon” but not that these achieve this goal, which in our opinion is insufficient as requirement and have been also noted as unclear by Dubois (2017b).
